# Integrated gut microbiota and metabolomic profiling reveals key associations between amino acid levels and gut microbial composition in patients with obesity

**DOI:** 10.3389/fnut.2025.1648469

**Published:** 2025-10-24

**Authors:** Musen Qi, Li Wang

**Affiliations:** ^1^Liaoning University of Traditional Chinese Medicine, Shenyang, China; ^2^The Affiliated Hospital of Liaoning University of Traditional Chinese Medicine, Shenyang, China

**Keywords:** obesity, gut microbiota, metabolomics, amino acids, organic acids

## Abstract

**Introduction:**

Obesity is an increasingly serious global health concern and is closely associated with gut dysbiosis and metabolic imbalance. Despite the considerable research conducted on the gut microbiota and metabolism over recent years, studies focusing on their correlation with obesity remain limited. In this study, we sought to characterize the gut microbiota and serum metabolic profiles of patients with obesity, aiming to identify potential biomarkers and therapeutic targets for this condition, and explore possible links between altered amino acid levels and gut microbial composition in its pathophysiology. The findings may offer novel insights into obesity prevention and treatment through microbiota modulation or amino acid regulation.

**Methods:**

Forty adult volunteers with obesity (BMI = 30.9 ± 2.9 kg/m^2^) who met the diagnostic criteria were enrolled in this study. Pregnant or lactating women and individuals with severe comorbidities were excluded. The control group comprised 20 subjects with normal weight (BMI = 21.9 ± 1.7 kg/m^2^) and without metabolic disorders, recruited from among outpatients during the same period and matched for age and sex. Fecal microbiota profiling was performed using 16S rRNA sequencing. DNA was extracted from stool samples, and the V3–V4 region was amplified and sequenced on the Illumina platform. After rigorous quality control (QC) and chimera removal, effective tags were clustered into Operational Taxonomic Units (OTUs) based on sequence similarity. Alpha and beta diversity and intergroup differential abundance were assessed, with statistical significance determined by Welch's *t*-test. Serum metabolomic analysis was performed using standardized sample preparation and QC procedures, followed by LC–MS/MS-based targeted and untargeted metabolomics. Calibration curves with *R*^2^ > 0.99 were established, and relative metabolite concentrations were calculated from peak areas. In total, 28 amino acid metabolites were quantified and used for subsequent statistical analysis.

**Results:**

Significant differences in microbial composition were observed across multiple taxonomic levels between the controls and patients with obesity. At the phylum level, Proteobacteria was enriched in the obesity group (AUC = 0.709, 95% CI: 0.569–0.848; *p* = 0.006). At the class level, Gammaproteobacteria (AUC = 0.712, 95% CI: 0.573–0.852; *p* = 0.009) and Erysipelotrichia (AUC = 0.614, 95% CI: 0.471–0.757; *p* = 0.02) were found to be enriched in obesity. At the order level, enrichment was observed for Enterobacteriales (AUC = 0.734, 95% CI: 0.597–0.871; *p* = 0.008) and Erysipelotrichia (AUC = 0.614, 95% CI: 0.471–0.757; *p* = 0.029). At the family level, Enterobacteriaceae (AUC = 0.614, 95% CI: 0.471–0.757; *p* = 0.003) showed enrichment in obesity. Finally, at the genus level, *Escherichia-Shigella* (AUC = 0.71, 95% CI: 0.565–0.855; *p* = 0.028) was enriched in obesity, while at the species level, *Bacteroides fragilis* (AUC = 0.733, 95% CI: 0.593–0.873; *p* = 0.016) and *Parabacteroides distasonis* (AUC = 0.61, 95% CI: 0.466–0.754; *p* = 0.033) were noted to be enriched. Metabolomic analysis revealed that in patients with obesity, the abundance of carnosine (log2FC = 1.16, FDR = 0.0016, VIP = 0.707), creatinine (log2FC = 0.21, FDR = 0.0009, VIP = 2.02), and cystine (log2FC = 0.55, FDR = 0.009, VIP = 1.47) was significantly increased compared with that in the controls; in contrast, that of ornithine (log2FC = −0.59, FDR = 0.0009, VIP = 1.19), citrulline (log2FC = −0.59, FDR = 0.0003, VIP = 0.707), glycine (log2FC = −0.54, FDR = 0.0003, VIP = 1.41), and serine (log2FC = −0.38, FDR = 0.0019, VIP = 1.62) was significantly decreased. This suggested that these metabolites may have potential as early diagnostic biomarkers for obesity.

**Conclusions:**

Obesity is associated with coordinated shifts in specific gut taxa and serum metabolites, with measurable effect sizes and strong discriminatory performance. Modulating amino acid levels or gut microbiota composition may represent a promising strategy for obesity prevention and treatment.

## 1 Introduction

Obesity is a chronic metabolic disease resulting from a combination of genetic predisposition, poor dietary habits, and other contributing factors. It is characterized by excessive fat accumulation and abnormal weight gain, with Body Mass Index (BMI) being the most commonly used measure for diagnosing this condition. Based on the guidelines of the Working Group on Obesity in China, obesity in Chinese adults is defined as a BMI ≥ 28 kg/m^2^. This threshold is lower than the WHO international standard (BMI ≥ 30 kg/m^2^) due to ethnic differences in body composition and metabolic responses. The prevalence of obesity is steadily increasing and is closely associated with the development of conditions such as cancer (1), hypertension, and type 2 diabetes (2). The primary cause of obesity is an imbalance between energy intake and expenditure, with simple obesity accounting for approximately 90% of all cases (3, 4).

The gut microbiota is increasingly recognized as a key determinant of human health (5). It is now known to be an essential component of the intestinal microecosystem, playing a vital role in the regulation of host nutrient absorption, the maintenance of the intestinal mucosal barrier, and the modulation of immune functions (6). Studies have shown that a stable gut microbiota is crucial for preserving intestinal function and overall health. Changes in the abundance of specific microbial populations can lead to an increase in the number of pathogenic bacteria while simultaneously decreasing that of beneficial ones, potentially leading to the development of obesity. Adipose tissue can release inflammatory cytokines such as tumor necrosis factor-alpha (TNF-α), interleukin-1 beta (IL-1β), and IL-6, thereby triggering systemic inflammatory responses (7). Obesity is increasingly recognized not only as a metabolic disorder but also as a state of chronic low-grade systemic inflammation, which may influence susceptibility to various diseases, including respiratory conditions (8). This gives rise to a complex “microbiota–metabolism–inflammation” interaction network, which is considered a key endogenous mechanism in the pathogenesis of obesity. Alterations in the composition, diversity, relative abundance, and functional pathways of gut microbiota may be important contributors to obesity in adults (9).

In the study of the biological processes underlying obesity, metabolomics has attracted considerable attention due to its ability to dynamically reflect the metabolic characteristics of an organism. Metabolomics is a relatively novel omics approach with high application value as a systematic tool for investigating the end-products of biological activities, enabling the comprehensive analysis of changes in metabolic pathways under both physiological and pathological conditions (10). Commonly used analytical metabolomic platforms include mass spectrometry (MS), nuclear magnetic resonance, liquid chromatography–mass spectrometry (LC-MS), and gas chromatography–mass spectrometry.

LC-MS is characterized by high sensitivity, broad detection coverage, and low sample volume requirements. Consequently, this platform has become an important tool for studying the metabolic characteristics of obesity. Quantitatively analyzing metabolites in biological samples can help establish correlations between physiological changes and metabolic alterations.

Targeted metabolomics focuses on the detection and analysis of specific metabolites or metabolic pathways, allowing for an in-depth exploration of the mechanisms of action of drugs within defined biochemical routes (11). This approach enables the isolation of metabolites of interest, thereby reducing interference from other highly abundant compounds (12). This, in turn, allows for more precise quantification and the generation of well-defined and complementary datasets for biological interpretation. It has evolved into several specialized branches, including functional, carbohydrate, lipid, and enzyme metabolomics, along with studies based on specific small molecules or metabolic pathways, such as those associated with nucleotide metabolism. Functional metabolomics is employed to uncover molecular mechanisms underlying interactions between disease-responsive biomarkers and functional compounds (secondary metabolites). Additionally, it can contribute to revealing how herbal-derived compounds block the biosynthesis of key disease-related molecules and modulate pathogenic biochemical interactions. This approach supports disease discovery and treatment, offering a modern framework that supports the systemic therapeutic characteristics of traditional Chinese medicine (TCM) (13).

Integrating metabolomics with obesity research, particularly in combination with gut microbiota profiling, can allow the identification of distinct metabolic patterns in different obesity phenotypes at the microscopic level as well as the discovery of potential diagnostic biomarkers and therapeutic targets. In addition to offering novel perspectives for understanding the mechanisms underlying the pathology of obesity, such an integrated strategy also provides a theoretical foundation and technical support for the development of personalized treatment strategies, as well as identifying novel targets and approaches for the future clinical diagnosis and management of obesity.

## 2 Materials and methods

### 2.1 Materials

#### 2.1.1 Fecal collection

The spoon provided with the fecal collection cup was used to collect the innermost portion of the stool sample (no less than 2 g). The sample was placed in a sterile EP tube, labeled with a serial number, and preserved in dry ice. The samples were subsequently stored at −80 °C within 2 h for further processing.

#### 2.1.2 Serum collection for metabolomic analysis

Venous blood (5 mL) was collected from each subject, labeled, and centrifuged at 3,000 rpm for 10 min at 4 °C. After centrifugation, 1 mL of the supernatant, containing serum, was equally divided into two EP tubes. To ensure the stability and quality of the serum samples, they were immediately preserved in dry ice and transferred to a −80 °C freezer within 2 h for subsequent use.

### 2.2 Inclusion and exclusion criteria

#### 2.2.1 Inclusion criteria

Participants were included if they (1) met the diagnostic criteria for obesity, defined as BMI ≥ 28 kg/m^2^; (2) were able to cooperate with blood and stool sampling and voluntarily agreed to participate in the study; (3) were aged between 18 and 80 years, regardless of gender; and (4) were fully conscious and capable of independently signing the informed consent form.

#### 2.2.2 Exclusion criteria

The criteria for exclusion included the following: (1) Pregnant or lactating women; (2) presenting with severe cardiovascular, cerebrovascular, pulmonary, hepatic, renal, or other major primary diseases, including complicated cardiovascular conditions or severe hepatic/renal insufficiency; (3) systemic antibiotic use within the past 3 months; (4) probiotic, prebiotic, or synbiotic supplementation within the past 4 weeks; (5) the use of immunosuppressant or corticosteroid therapy within the past 3 months; (6) diagnosed with a gastrointestinal disease, including inflammatory bowel disease or celiac disease, or a history of major gastrointestinal surgery within the past 6 months; (7) history of alcoholism, use of psychoactive substances, drug abuse, or drug dependence; (8) recent use of weight-loss drugs or health supplements; (9) hospitalization within the past month; (10) poor compliance with or inability to complete the study protocol; or (11) not meeting the inclusion criteria.

#### 2.2.3 Selection of healthy controls

Healthy controls were recruited from among individuals attending outpatient clinics during the same period. The inclusion criteria were BMI 18.5–24 kg/m^2^, the absence of underlying metabolic diseases, similar age (±3 years) and gender distribution to the obese group, and age ≥18 years. All controls voluntarily participated in the study and provided signed and informed consent.

### 2.3 Experimental procedure

#### 2.3.1 Gut microbiota analysis

Clinical data and fecal samples were collected from all participants, including 40 patients with obesity (mean BMI 30.9 ± 2.9 kg/m^2^; mean age 45.4 ± 17.9 years) and 20 healthy controls (mean BMI 21.9 ± 1.7 kg/m^2^); mean age 43.4 ± 14.9 years). DNA was extracted from the fecal samples, and the V3–V4 region of the 16S rRNA gene was amplified by PCR. Sequencing libraries were constructed and sequenced on the Illumina platform. Data analysis included microbial abundance, diversity, and community composition.

##### 2.3.1.1 Sequencing depth and data processing

Raw reads were filtered to remove low-quality sequences. Paired-end reads were then merged to generate tags, and chimeric sequences were removed. Operational Taxonomic Units (OTUs) were clustered at a 97% similarity threshold. Alpha and beta diversity analyses, differential taxa determination, and visualization were performed using QIIME2 (v.2021) and R software (v.4.1.2). Appropriate statistical tests, including Welch's *t*-test, were applied, with *p* < 0.05 considered statistically significant.

#### 2.3.2 Metabolomics analysis

Serum samples were collected from all 60 participants (40 patients with obesity and 20 healthy controls). Based on clinical manifestations and TCM syndrome classification, obese patients were further categorized into Group A, characterized by liver qi stagnation and spleen deficiency, and Group B, typified by blood stasis and phlegm retention. Both targeted and non-targeted metabolomics analysis were undertaken. For metabolomic profiling, samples were thawed at 4 °C, and 100 μL of serum was extracted with 1 mL of pre-cooled 80% methanol. After vortexing, sonication, and precipitation, the supernatant was concentrated, reconstituted in 50% methanol, and centrifuged for LC–MS analysis. To monitor system stability, QC samples were prepared by pooling equal aliquots of all serum samples. These were injected at the beginning and end of the sequence, as well as every 12 samples during the sequence. Metabolites were analyzed using an AB SCIEX 5500 QTRAP mass spectrometer in positive ion mode with multiple reaction monitoring (MRM). Chromatographic separation was performed at 40 °C and a flow rate of 0.3 mL/min using a binary mobile phase (0.1% formic acid with 10 mM ammonium formate in water, and acetonitrile with 0.1% formic acid). Data were processed using MultiQuant software, and metabolite quantification was achieved against amino acid standards using external calibration (*R*^2^ > 0.99).

##### 2.3.2.1 QC strategy

Pooled QC samples were prepared by mixing equal aliquots from all samples. The QC samples were periodically injected to monitor instrument stability. The relative standard deviation (RSD) of detected metabolites in QC samples was < 30%, and a principal component analysis (PCA) confirmed tight clustering, thereby validating workflow reliability.

##### 2.3.2.2 Compound identification and quantification

Metabolites were identified by comparing retention times and mass spectra with 42 authentic amino acid and derivative standards. Calibration curves were established at five concentrations (0.02, 0.05, 0.2, 0.5, and 1 μg/mL) with *R*^2^ > 0.99. A total of 28 amino acids and derivatives were quantified. Significantly altered metabolites and related pathways were further analyzed to support an investigation into obesity-related mechanisms.

### 2.4 Statistical methods

The data relating to gut microbiota analysis were analyzed using SPSS 26.0. For categorical variables, the chi-square test was applied. Normality was assessed with the Shapiro–Wilk test. Normally distributed data were expressed as means ± SD and compared using independent-samples *t*-tests; non-normally distributed data were analyzed using the Mann–Whitney U-test. Alpha diversity indices (Sobs, Chao1, ACE, Simpson, Shannon, Pielou, PD-tree) were calculated. Beta diversity and species abundance were visualized using R software, with Welch's *t*-test for taxonomic comparisons. *p* < 0.05 was considered statistically significant.

MS-DIAL software was employed for metabolomics data processing, including peak alignment, retention time correction, and peak area extraction. Metabolite identification was performed based on accurate mass matching (mass tolerance < 10 ppm) and MS/MS spectral matching (mass tolerance < 0.01 Da) against multiple public databases, including MDB, MassBank, and GNPS, as well as an in-house standard compound library (BP-DB). Ion features missing in >50% of samples within a group were excluded from subsequent analyses. Positive and negative ion datasets were normalized by total peak area, integrated, and submitted to pattern recognition using Python software. Data were preprocessed using unit variance (UV) scaling before statistical analysis. To ensure system stability, QC samples were evaluated using both base peak chromatogram (BPC) comparison and PCA of all the samples.

### 2.5 ROC analysis of differential metabolites

Receiver Operating Characteristic (ROC) curve analysis was used to evaluate the diagnostic potential of significantly altered metabolites. Logistic regression was applied, with group classification (obese *vs*. healthy) as the dependent variable and the relative abundances of metabolites as independent variables. Predicted probabilities from the model were used to plot ROC curves and calculate the area under the curve (AUC). Five-fold cross-validation was applied to ensure model stability. Metabolites with an AUC of >0.7 with good reproducibility were identified as potential biomarkers. All ROC analyses were conducted using R software.

## 3 Results

### 3.1 Correlation between obesity and the gut microbiota

**Figure 1:**
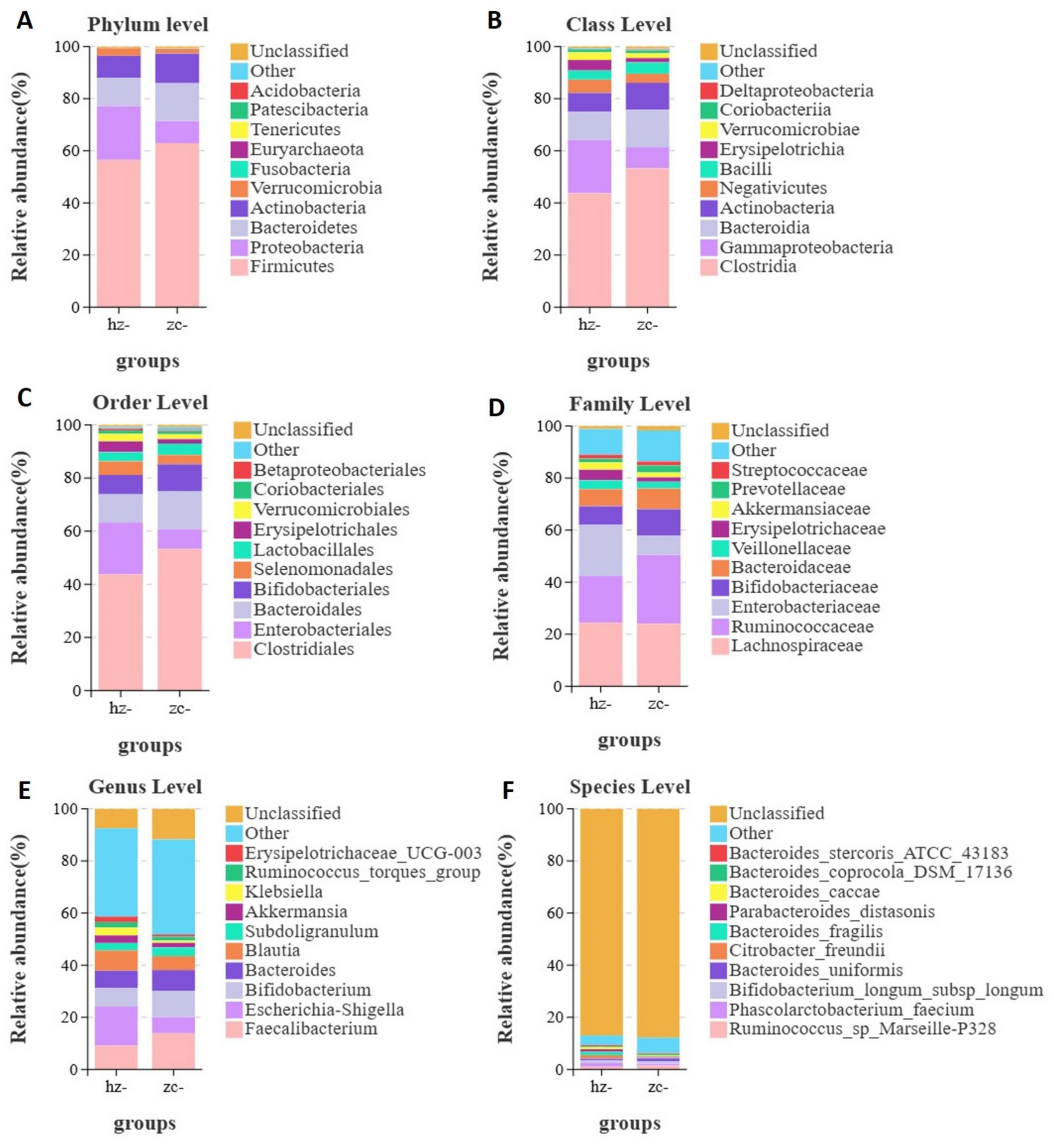
Differences in gut microbiota composition between patients with obesity (hz) and normal controls (zc). Relative abundance profiles of the gut microbiota at various taxonomic levels. Significant differences in abundance were observed in phyla such as Proteobacteria, Firmicutes, and Bacteroidetes. Differences were assessed using Welch's *t*-test for taxonomic comparisons; an adjusted *p*-value of < 0.05 was considered significant. **(A)** Phylum level; **(B)** Class level; **(C)** Order level; **(D)** Family level; **(E)** Genus level; **(F)** Species level.

Studies have shown that the composition of the gut microbiota in individuals with obesity differs significantly from that in healthy controls across various taxonomic levels (phylum, class, order, family, genus, and species). At the phylum level (Figure 1A), the relative abundance of Proteobacteria (AUC = 0.709, 95% CI: 0.569–0.848; *p* = 0.006) was higher in the obesity group than in the normal control group, suggesting that this phylum was dominant within the gut microbiota of the former group. In contrast, the relative abundance of Firmicutes (*p* = 0.245), Bacteroidetes (*p* = 0.148), and Actinobacteria (*p* = 0.512) was decreased in the obesity group, although the differences were not statistically significant (all *p* < 0.05). Notably, these phyla are also considered core components of the gut microbial community. At the class level (Figure 1B), the relative abundance of Gammaproteobacteria (AUC = 0.712, 95% CI: 0.573–0.852; *p* = 0.009) and Erysipelotrichia (AUC = 0.614, 95% CI: 0.471–0.757; *p* = 0.02) was higher in the obesity group than in the normal control group, whereas that of Verrucomicrobiae (*p* = 0.863) and Coriobacteriia (*p* = 0.939) was lower, but again not significantly (all *p* < 0.05). These phyla are also considered core components of the gut microbial community. At the order level (Figure 1C), meanwhile, compared with normal controls, the relative abundances of Enterobacteriales (AUC = 0.734, 95% CI: 0.597–0.871; *p* = 0.008) and Erysipelotrichia (AUC = 0.614, 95% CI: 0.471–0.757; *p* = 0.029) were significantly increased in patients with obesity, implying that they were predominant within the gut microbiota of this population. At the family level (Figure 1D), the dominant families included Lachnospiraceae (*p* = 0.05), Ruminococcaceae (*p* = 0.038), Enterobacteriaceae (*p* = 0.003), Bifidobacteriaceae (*p* = 0.512), and Bacteroidaceae (*p* = 0.497). Notably, the elevated abundance of Enterobacteriaceae (AUC = 0.614, 95% CI: 0.471–0.757) in the obesity group may contribute to gut microbiota imbalance and, thus, obesity development. At the genus level (Figure 1E), the five most abundant genera were *Escherichia-Shigella* (*p* = 0.028), *Faecalibacterium* (*p* = 0.143), *Bifidobacterium* (*p* = 0.503), *Bacteroides* (*p* = 0.483), and *Blautia* (*p* = 0.08). Among them, *Escherichia-Shigella* (AUC = 0.71, 95% CI: 0.565–0.855) was significantly less abundant in the obesity group than in the control group, potentially leading to dysbiosis and, ultimately, obesity. At the species level (Figure 1F), the top five species in terms of abundance were *Phascolarctobacterium faecium* (*p* = 0.333), *Ruminococcus* sp. Marseille-P328 (*p* = 0.226), *Bifidobacterium longum* subsp. *longum* (*p* = 0.923), *Parabacteroides distasonis* (*p* = 0.033), and *Bacteroides fragilis* (*p* = 0.016). Among these, *Bacteroides fragilis* (AUC = 0.733, 95% CI: 0.593–0.873; *p* = 0.016) and *Parabacteroides distasonis* (AUC = 0.61, 95% CI: 0.466–0.754; *p* = 0.033) were significantly enriched in the obesity group, highlighting their potential as obesity-specific indicators. The other three species showed a notable decline in abundance. Combined, these results suggested that a link may exist between gut microbial imbalance and obesity onset and progression.

#### 3.1.1 Alpha diversity analysis

In studies on gut microbiota ecology, alpha diversity reflects the richness, evenness, and overall diversity of microbial communities within a sample. In this study, we assessed the differences between the obesity and control groups using several indices, including the Sobs, Chao1, ACE, Shannon, Simpson, PD-tree, and Pielou indices. The results demonstrated that, across all indices, the values observed in the obesity group were consistently lower than those in the healthy control group. This indicated that individuals with obesity exhibit reduced microbial diversity and evenness in their gut microbiota. These findings suggested that a strong correlation exists between gut microbial diversity and the development of obesity.

**Figure 2:**
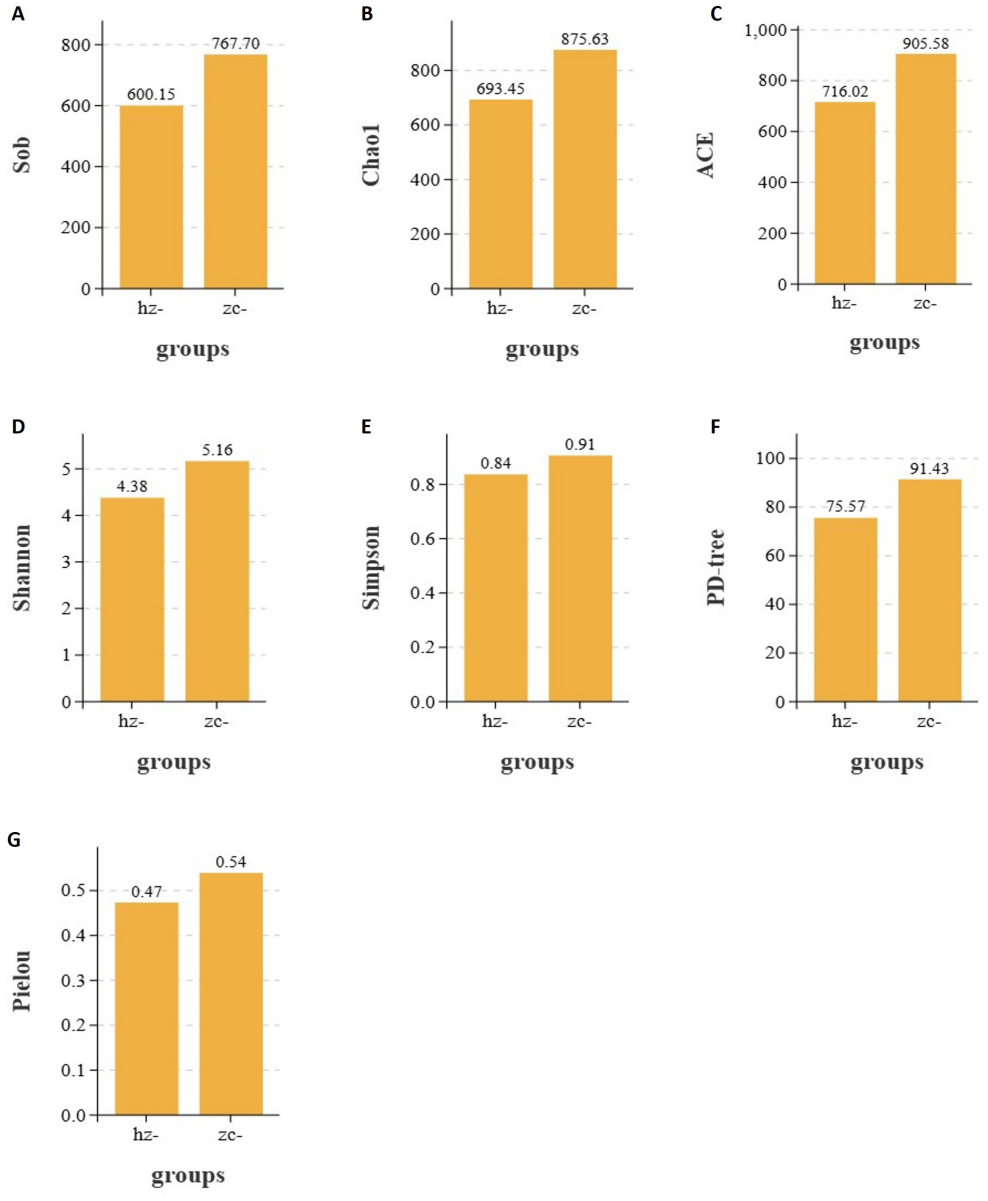
Alpha diversity indices of the gut microbiota in the obesity (hz) and control groups (zc). Sobs, Chao1, ACE, Shannon, Pielou, and PD-tree index values were significantly lower in the obesity group than in the control group (*p* < 0.05), whereas the Simpson index remained unchanged (*p* > 0.05). This indicated that richness, evenness, and phylogenetic diversity were reduced in patients with obesity. **(A)** Sobs; **(B)** Chao1; **(C)** ACE; **(D)** Shannon; **(E)** Simpson; **(F)** PD tree; **(G)** Pielou.

As shown in Figures 2A–C, the Sobs, Chao1, and ACE indices of the obesity group were significantly lower than those of the control group (*p* < 0.05), indicating that microbial richness was markedly reduced in patients with obesity compared with that in healthy controls (Table 1). As shown in Figure 2G, the Pielou index in the obesity group was significantly lower than that in the control group (*p* < 0.05), implying that the evenness of the gut microbiota was lower in the former population (Table 1). In addition, as shown in Figures 2D, E, both the Shannon and Simpson indices were lower in the obesity group, with the difference in the former (*p* < 0.05), but not the latter (*p* > 0.05), reaching statistical significance (Table 1). Furthermore, the PD-tree index was significantly lower in the obesity group than in the control group (*p* < 0.05) (Figure 2F), indicating that phylogenetic diversity had undergone a notable decline in patients with obesity (Table 1). Taken together, these results demonstrated that the gut microbiota of patients with obesity displays reduced richness, evenness, and overall diversity relative to healthy controls, suggesting that microbial community structure is disrupted in obesity.

**Table 1:** Alpha-diversity in obese patients and healthy controls.

**Index**	***p***
Sob	0.000016^**^
Chao1	0.0000029^**^
ACE	0.0000028^**^
Pielou	0.0026^*^
Simpson	0.0108
Shannon	0.0006^**^
PD-tree	0.0001^*^

Statistical analysis of alpha-diversity differences between patients with obesity and healthy controls. Welch's *t*-test was applied for group comparisons, with *p*-values shown.

^*^p < 0.05 indicates significant differences, ^**^p < 0.01 indicates highly significant differences.

#### 3.1.2 Beta diversity analysis

**Figure 3:**
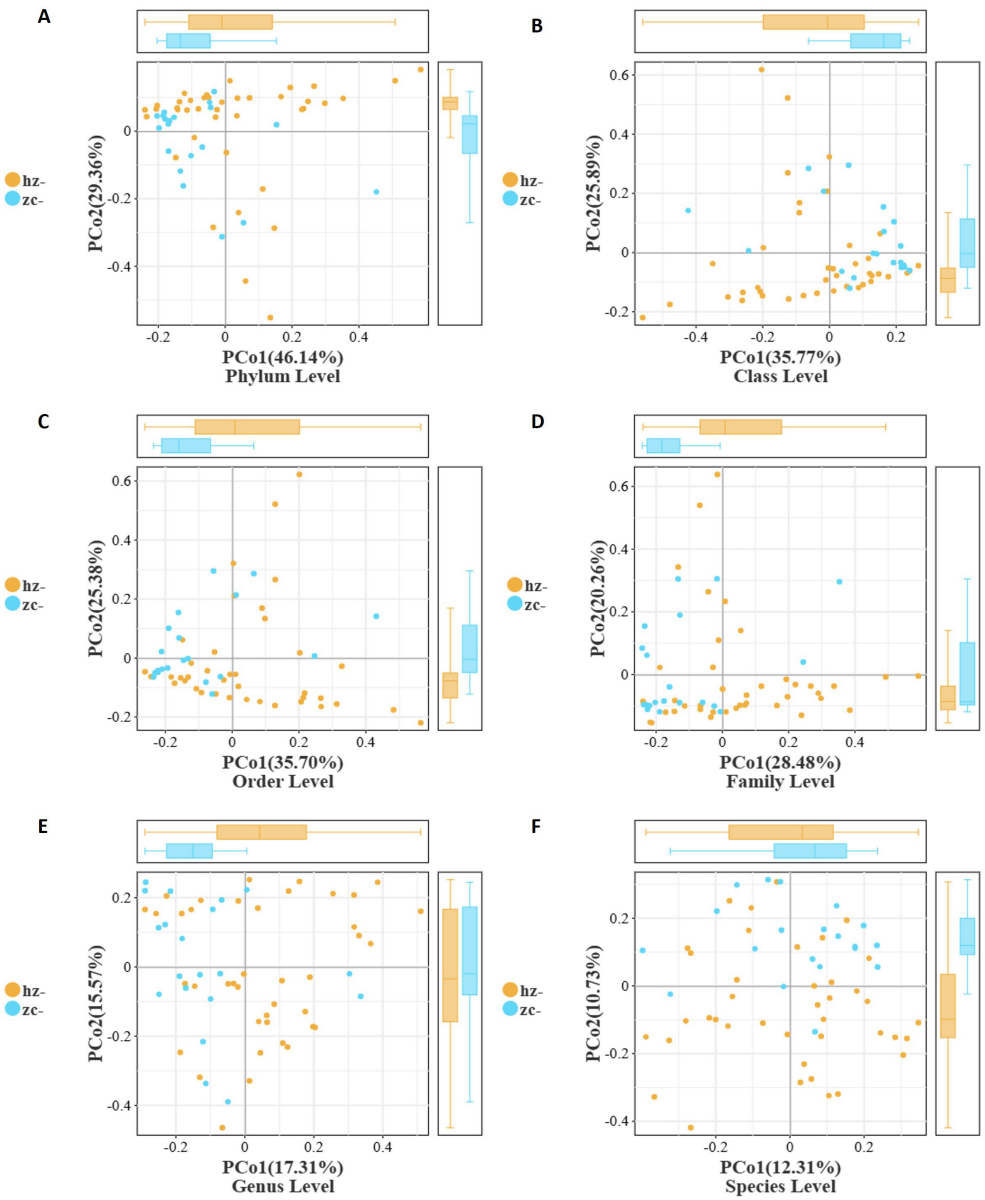
Beta-diversity of the gut microbiota between patients with obesity (hz) and healthy controls (zc) based on Bray–Curtis distances. PCoA plots show a clear separation between the two groups from the phylum to the order level, with the obesity group exhibiting greater heterogeneity. Boxplots further confirmed significant differences in PCo1/PCo2 distributions, while separation became less pronounced at finer taxonomic levels. **(A)** Phylum Level; **(B)** Class Level; **(C)** Order Level; **(D)** Family Level; **(E)** Genus Level; **(F)** Species Level.

To evaluate the overall structural differences of the gut microbiota between patients with obesity and healthy controls, we performed principal coordinate analysis (PCoA) based on Bray–Curtis distances across six taxonomic levels (phylum, class, order, family, genus, and species) (Table 2). As shown in Figure 3A, at the phylum level, a clear separation was observed between the two groups. PCo1 (46.14%) effectively distinguished patients with obesity from healthy controls, with the obesity group exhibiting greater intra-group dispersion. A similar pattern was evident at the class and order levels (PCo1 = 35.77% and 35.70%, respectively). As illustrated in Figures 3B, C, the obesity group tended to cluster along the positive axis of PCo1 with a wider interquartile range, while the control group was mainly concentrated on the negative axis. Notably, the contribution of PCo2 to group differentiation increased from the class level onward, with the control group showing consistently higher medians and broader distributions along this axis (Figure 3B). Additionally, at the family and genus levels, as the variance explained by PCo1 decreased (28.48% and 17.31%, respectively), the separation between the groups weakened (Figures 3D, E). Nevertheless, the obesity group maintained a persistent shift along the positive side of PCo1 and displayed greater internal heterogeneity, whereas the control showed an upward trend along PCo2 with a more dispersed distribution. At the species level (PCo1 = 12.31%, PCo2 = 10.73%), the overlap between the groups markedly increased, suggesting that at finer taxonomic resolution, differences became less pronounced and were mainly reflected in subtle changes in community positioning and dispersion rather than distinct clustering (Figure 3F).

**Table 2:** Beta-diversity across taxonomic levels in obese patients and healthy controls.

**Level**	***p***
Phylum	3.7293E−13^**^
Class	2.9752E−17^**^
Order	2.8466E−18^**^
Family	4.9629E−19^**^
Genus	6.3120E−14^**^
Species	4.2792E−16^**^

Statistical analysis of beta diversity differences between patients with obesity and healthy controls across taxonomic levels (phylum to species) based on Bray–Curtis distances. Welch's *t*-test was applied for group comparisons, with p-values shown.

^*^p < 0.05 indicates significant differences, ^**^p < 0.01 indicates highly significant differences.

Collectively, these findings indicated that, compared with healthy individuals, patients with obesity displayed significant differences in gut microbial community structure, which were most pronounced at higher taxonomic levels and primarily captured by PCo1. The obesity group consistently exhibited greater heterogeneity and a rightward shift on PCo1, while the control group showed a systematic elevation on PCo2 starting from the class level. These patterns suggested that obesity-related alterations in the gut microbiota are more robust and stable at higher taxonomic ranks, whereas at the species level, differences are diminished, primarily reflecting variability in community distribution.

Studies by Lehtonen H, Serkova N. J., and colleagues have shown that the gut microbiota composition of individuals with obesity differs significantly from that of healthy controls, with imbalances spanning multiple taxonomic levels from phylum to species (14, 15). We found that in patients with obesity, the Sobs, Chao1, ACE, Simpson, Shannon, Pielou, and PD-tree indexes were all markedly lower than those of healthy individuals, indicating that richness, evenness, and overall diversity of the gut microbiota were decreased in the former group. Notably, Proteobacteria were significantly enriched in individuals with obesity, while Firmicutes, Bacteroidetes, and Actinobacteria showed a non-significant decreasing trend in abundance. At the class and order levels, Gammaproteobacteria, Erysipelotrichia, and Enterobacteriales showed significant enrichment in the obesity group, potentially leading to intestinal homeostasis disruption. At the family and genus levels, the abundance of Enterobacteriaceae was markedly elevated in the obesity group, whereas that of *Escherichia-Shigella* was significantly reduced. At the species level, *Bacteroides fragilis* and *Parabacteroides distasonis* were more abundant in individuals with obesity, indicating that they may serve as obesity-related microbial indicators.

In summary, we found notable differences in the distribution, richness, and evenness of the gut microbiota between individuals with obesity and healthy controls. Significant variations were observed in taxonomic groups such as Proteobacteria, Clostridiales, Lachnospiraceae, Enterobacteriaceae, and *Escherichia-Shigella*, highlighting their potential to serve as biomarkers for obesity as well as targets for disease prevention, diagnosis, and treatment.

### 3.2 Analysis of the correlation between obesity and changes in the metabolome

**Figure 4:**
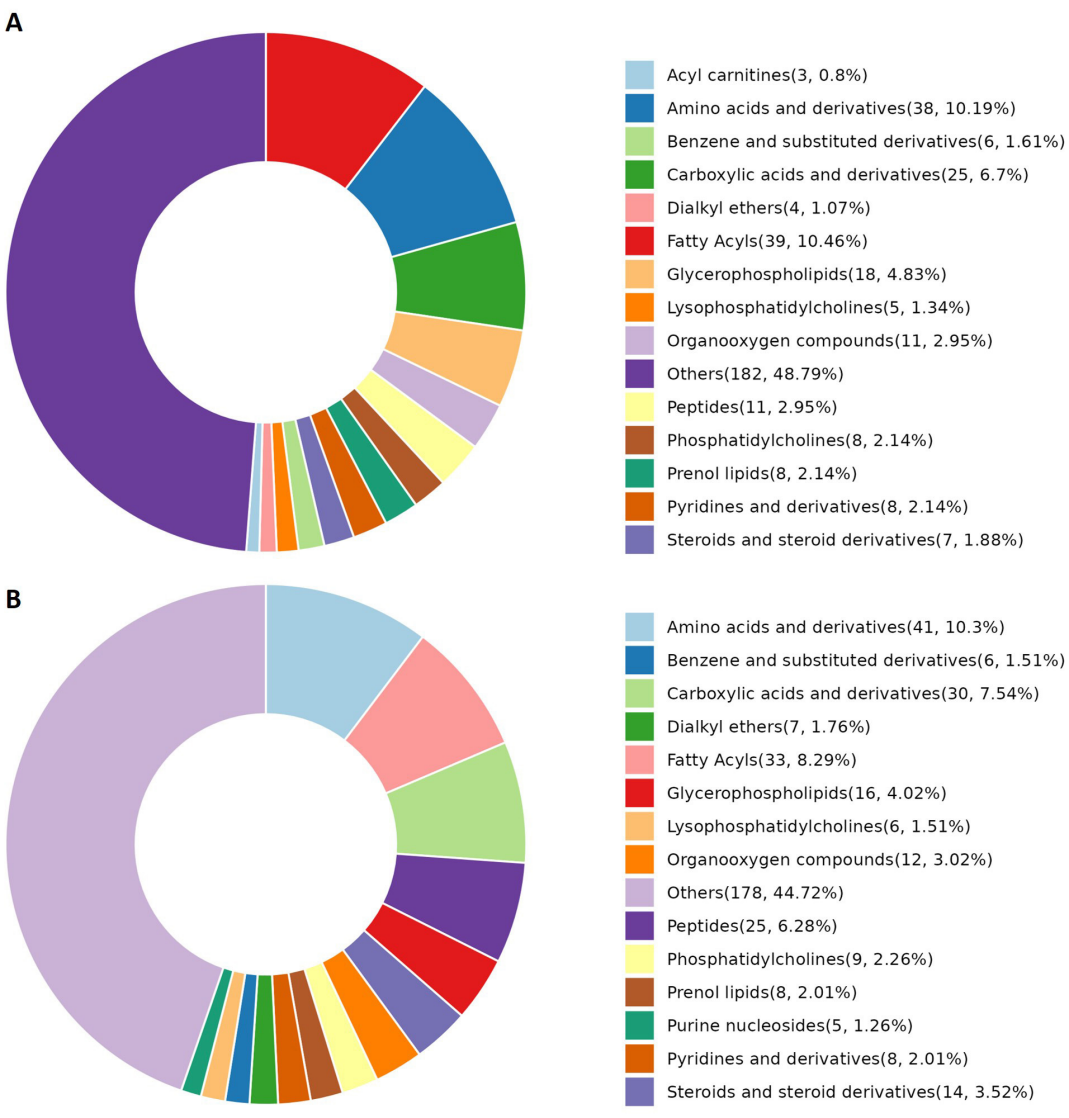
Metabolite categories displaying differential abundance between the obesity and control groups. **(A)** The relative abundance of differential metabolite categories between patients in Group A (characterized by liver qi stagnation and spleen deficiency) and healthy controls. **(B)** The relative abundance of differential metabolite categories between patients in Group B (characterized by blood stasis and phlegm retention) and healthy controls. The color blocks on the right indicate metabolite categories. Differential metabolites were identified using multivariate analysis with the criteria VIP > 1.0, |log2FC| ≥ 0.5, and FDR < 0.05.

**Figure 5:**
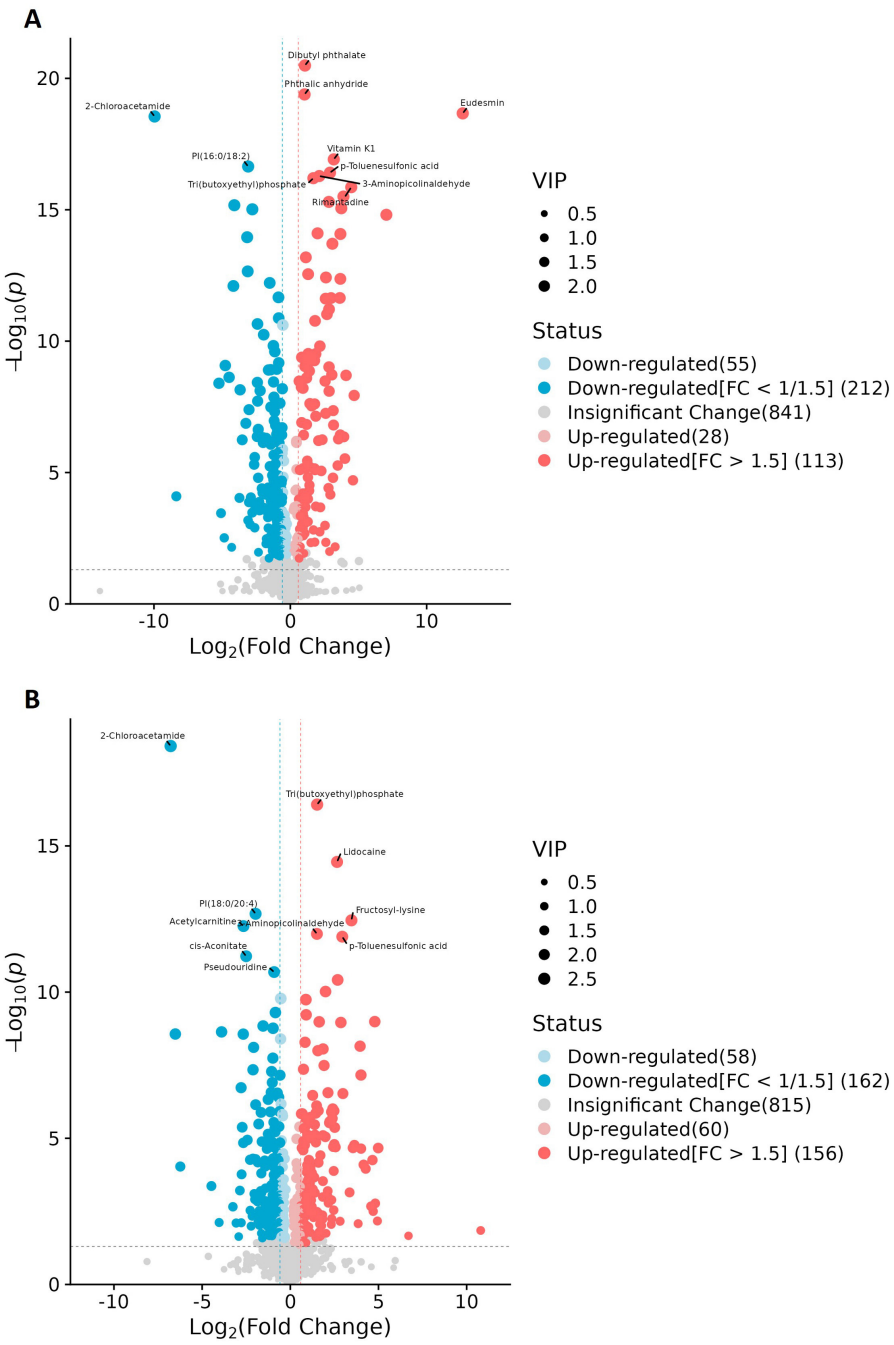
Volcano plots of the differential metabolites. **(A)** Patients in Group A (characterized by liver qi stagnation and spleen deficiency) vs. healthy controls. **(B)** Patients in Group B (characterized by blood stasis and phlegm retention) vs. healthy controls. Each dot represents a metabolite (*x*-axis: log2FC, *y*-axis: –log10p); dot size indicates VIP score. Blue and red denote down- and upregulated metabolites, respectively, while gray indicates no significant change.

Differences in the abundance of metabolites and their corresponding main metabolic pathways between the obesity and healthy control groups were analyzed using non-targeted LC-MS. Metabolomics studies have identified key metabolic pathways associated with obesity, including those associated with lipid, amino acid, glucose, phospholipid, and gut microbial metabolism (16–18). In this study, the metabolite categories showing the greatest differences in abundance between the obesity and control groups were amino acids and derivatives, fatty acyls, and carboxylic acids and derivatives (Figures 4A, B, 5A, B).

**Figure 6:**
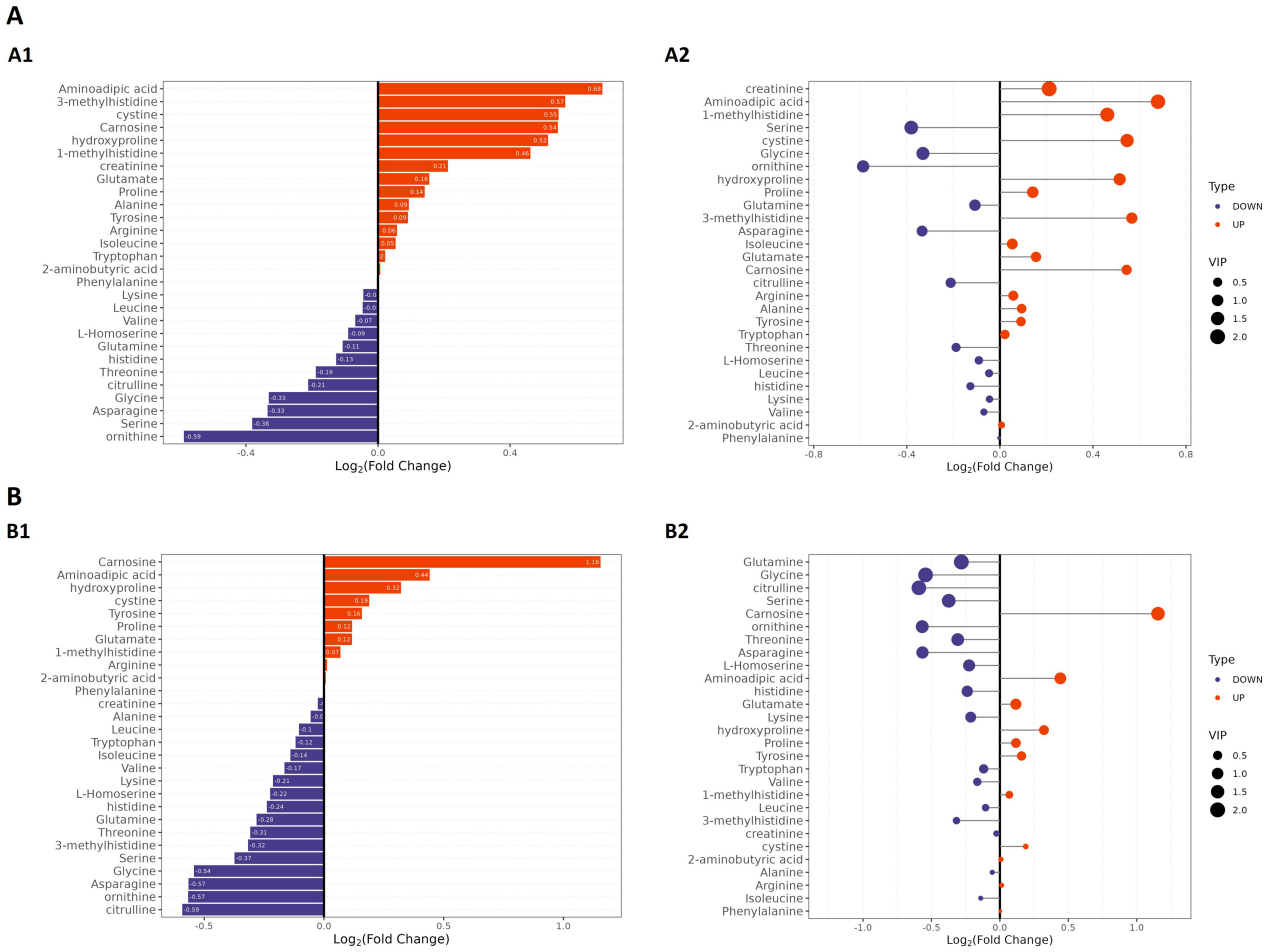
Targeted amino acid metabolomic profiling by LC–MS in patients with obesity and healthy controls. **(A)** Group A vs. controls. **(A1)** Bar plot of amino acids ranked by log2FC in Group A; the *x*-axis shows log2FC, with positive values indicating higher levels, and negative values indicating lower levels. **(A2)** Bubble plot of the same features; the *x*-axis shows log2FC, bubble size reflects VIP, and color denotes up- or downregulation. **(B)** Group B vs. controls. **(B1)** Bar plot of amino acids ranked by log2FC for Group B. **(B2)** Bubble plot as in **(A2)** for Group B. Differential metabolites were defined using VIP > 1.0, |log2FC| ≥ 0.5, and FDR < 0.05 as the criteria.

Given that the non-targeted metabolomic analysis showed that differential metabolites were predominantly enriched in amino acid metabolism pathways, we next undertook a targeted analysis of amino acid metabolites. Based on *a priori* thresholds (VIP > 1.0, |log2FC| ≥ 0.5, FDR < 0.05), amino acid-related alterations predominated across both comparisons (Group A or Group B vs. controls). In the comparison between Group A and the controls, significant increases in abundance were observed for aminoadipic acid (log2FC = 0.68), 3-methylhistidine (=0.57), cystine (=0.55), carnosine (=0.54), and hydroxyproline (=0.52), while ornithine contents were decreased (=-0.59). In the Group B vs. controls comparison, carnosine showed a marked increase in abundance (=1.16; highest VIP), whereas the abundances of citrulline (=-0.59), ornithine (=-0.57), asparagine (=-0.57), and glycine (=-0.54) were reduced. Together with concordant, albeit sub-threshold, shifts in serine (downregulation), proline, alanine, tyrosine, arginine, and glutamate (upregulation) levels, these findings indicated that Group B patients experienced an attenuation of the urea cycle/NO pathway and a reduction in one-carbon/glutathione capacity (downregulation of ornithine, citrulline, glycine, and serine), alongside intensified lysine catabolism/oxidative stress and extracellular-matrix remodeling (upregulation of aminoadipic acid and hydroxyproline). Furthermore, our data suggested that muscle protein turnover was increased in Group A patients, as reflected by increased 3-methylhistidine and creatinine levels, while the histidine/carnosine buffering/antioxidant response was accentuated in patients in Group B, as evidenced by the observed upregulation of carnosine and cystine contents, with carnosine showing higher discriminative weight. Collectively, increases in the abundance of aminoadipic acid, hydroxyproline, and carnosine, along with decreases in that of ornithine, citrulline, glycine, and serine, define a coherent amino acid signature distinguishing obesity in this cohort (Figures 6A1–B2).

#### 3.2.1 ROC analysis

**Figure 7:**
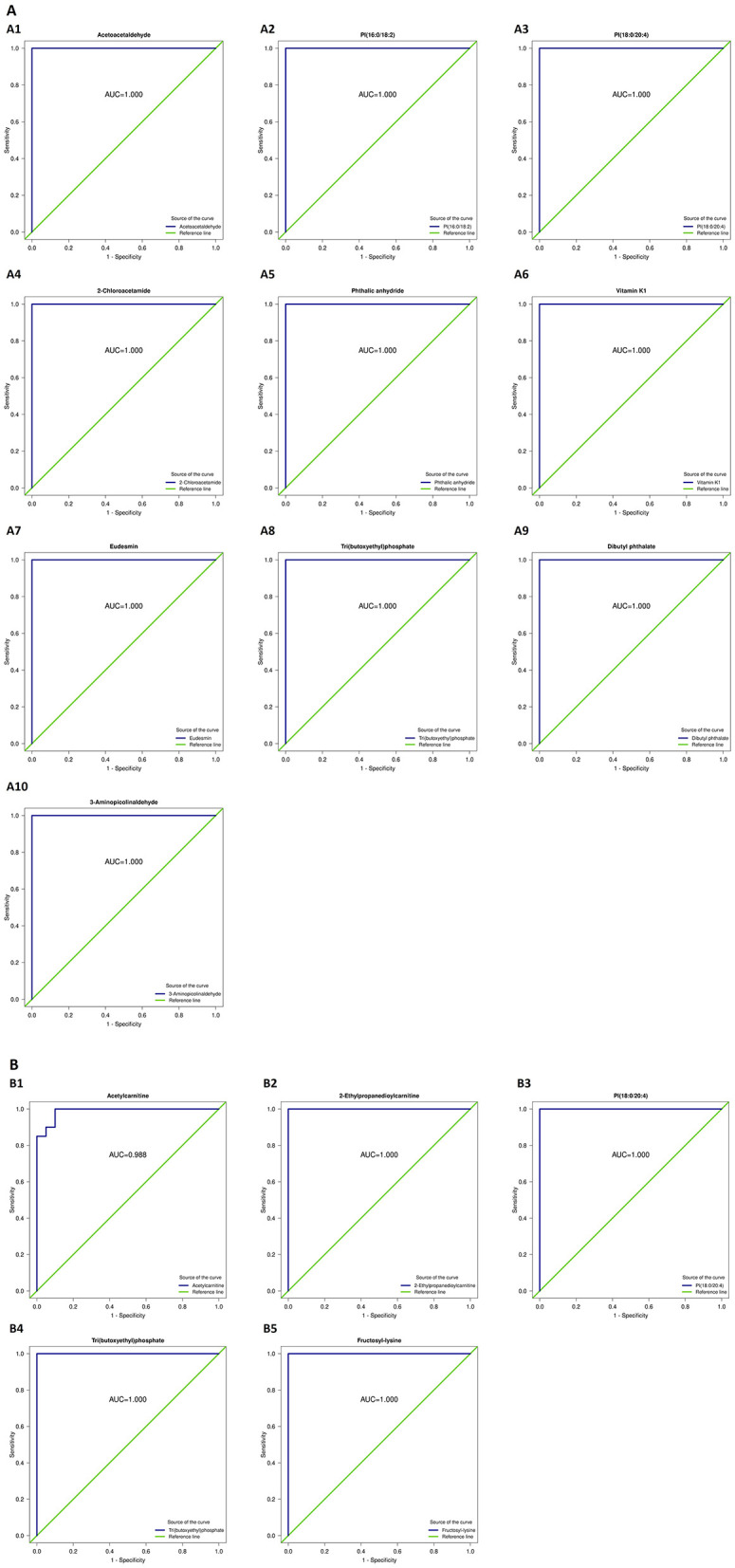
Receiver operating characteristic (ROC) curves of the differential metabolites capable of distinguishing patients with obesity from healthy controls. **(A1)** Acetoacetaldehyde; **(A2)** PI(16:0/18:2); **(A3)** PI(18:0/20:4); **(A4)** 2-Chloroacetamide; **(A5)** Phthalic anhydride; **(A6)** Vitamin K1; **(A7)** Eudesmin; **(A8)** Tri(butoxyethyl)phosphate; **(A9)** Dibutyl phthalate; **(A10)** 3-Aminoisobutyraldehyde; **(B1)** Acetylcarnitine; **(B2)** 2-Ethylpropanedioylcarnitine; **(B3)** PI(18:0/20:4); **(B4)** Tri(butoxyethyl)phosphate; **(B5)** Fructosyl-lysine.

ROC analysis of the differentially abundant metabolites identified substances such as dibutyl phthalate, phthalic anhydride, eucalyptol, vitamin K1, tripropylphosphate, glycine, 2-chloroacetamide, phosphatidylinositol PI(16:0/18:2), acetylcarnitine, PI(18:0/20:4), lysine, and 2-chloroacetamide as obesity-specific markers, capable of distinguishing between patients with obesity and healthy controls (Figure 7).

#### 3.2.2 Heatmap analysis

**Figure 8:**
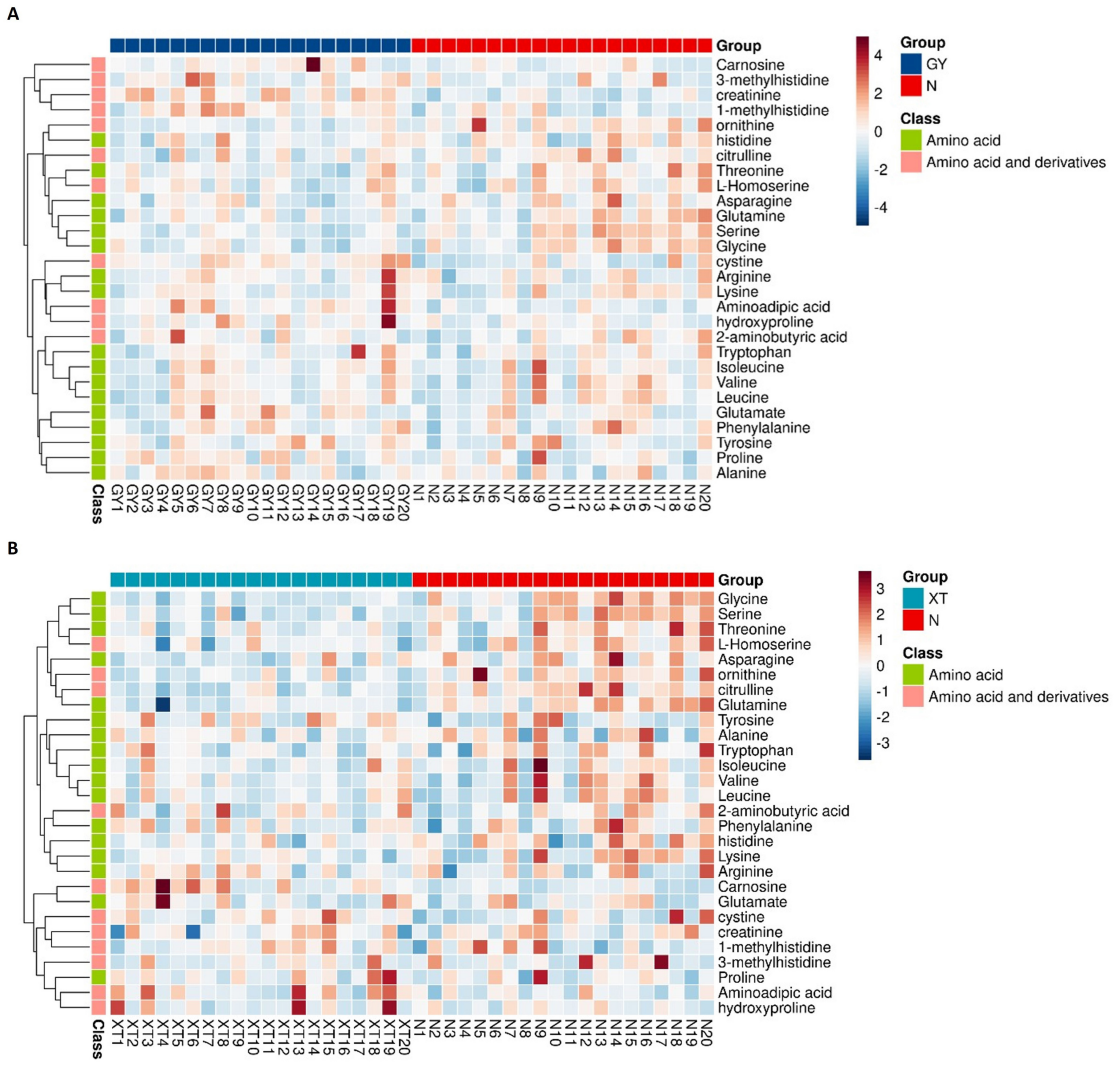
Heatmaps of targeted amino acid metabolomic profiles. **(A)** Group A vs. controls. Comparison of the targeted amino acid (and derivatives) metabolomic profiles between obesity cohort A (GY, *n* = 20) and healthy controls (*N, n* = 20). Rows are metabolites; columns are individual study participants. Values were row-standardized (*Z*-scores). Color scale: red denotes higher relative abundance, blue signifies lower relative abundance. The left dendrogram shows unsupervised hierarchical clustering; the side strip shows the chemical class (green: amino acid; pink: amino acid and derivatives). **(B)** Group B vs. controls. Comparison of the targeted amino acid (and derivatives) metabolomic profiles between obesity cohort B (XT, *n* = 20) and healthy controls (N, *n* = 20). Display conventions are identical to **(A)**.

Based on the quantitative profiling of 48 amino acids, heatmaps were generated for the two comparison groups (Group A or B vs. controls), providing sample-level confirmation of robust differences between patients with obesity and healthy controls. After *Z*-score standardization and unsupervised hierarchical clustering, patient samples were separated from control samples along the column dendrogram. As shown in Figure 8A, the abundance of carnosine, 3-methylhistidine, and creatinine was generally higher in Group A than in the control group, whereas that of ornithine, citrulline, glycine, and serine was lower. Histidine and L-homoserine contents also tended to decrease in Group A patients. Meanwhile, aminoadipic acid and hydroxyproline were enriched in a subset of patients. The same primary pattern was observed for the Group B vs. controls comparison, but with a more uniform increase in the abundance of carnosine; glycine, serine, citrulline, and ornithine levels were largely reduced in Group B patients; phenylalanine, tyrosine, and tryptophan displayed modest enrichment in a minority of patients; and asparagine and glutamine contents tended to be lower (Figure 8B). These heatmap patterns agreed with the targeted metabolomics results and indicated that both the urea cycle/NO and one-carbon/glutathione axes were downregulated in obesity (indicated by lower levels of ornithine, citrulline, glycine, and serine), whereas lysine degradation/oxidative stress and extracellular-matrix remodeling were enhanced (evidenced by higher aminoadipic acid, hydroxyproline, and carnosine abundance). Overall, the increases in the contents of carnosine, 3-methylhistidine, creatinine, aminoadipic acid, and hydroxyproline, coupled with decreases in those of ornithine, citrulline, glycine, and serine, define a coherent amino acid signature that not only provides evidence of lipid-metabolic imbalance and dysregulated inflammatory control in obesity, but also identifies potential biomarkers and therapeutic targets for this condition.

#### 3.2.3 KEGG analysis

**Figure 9:**
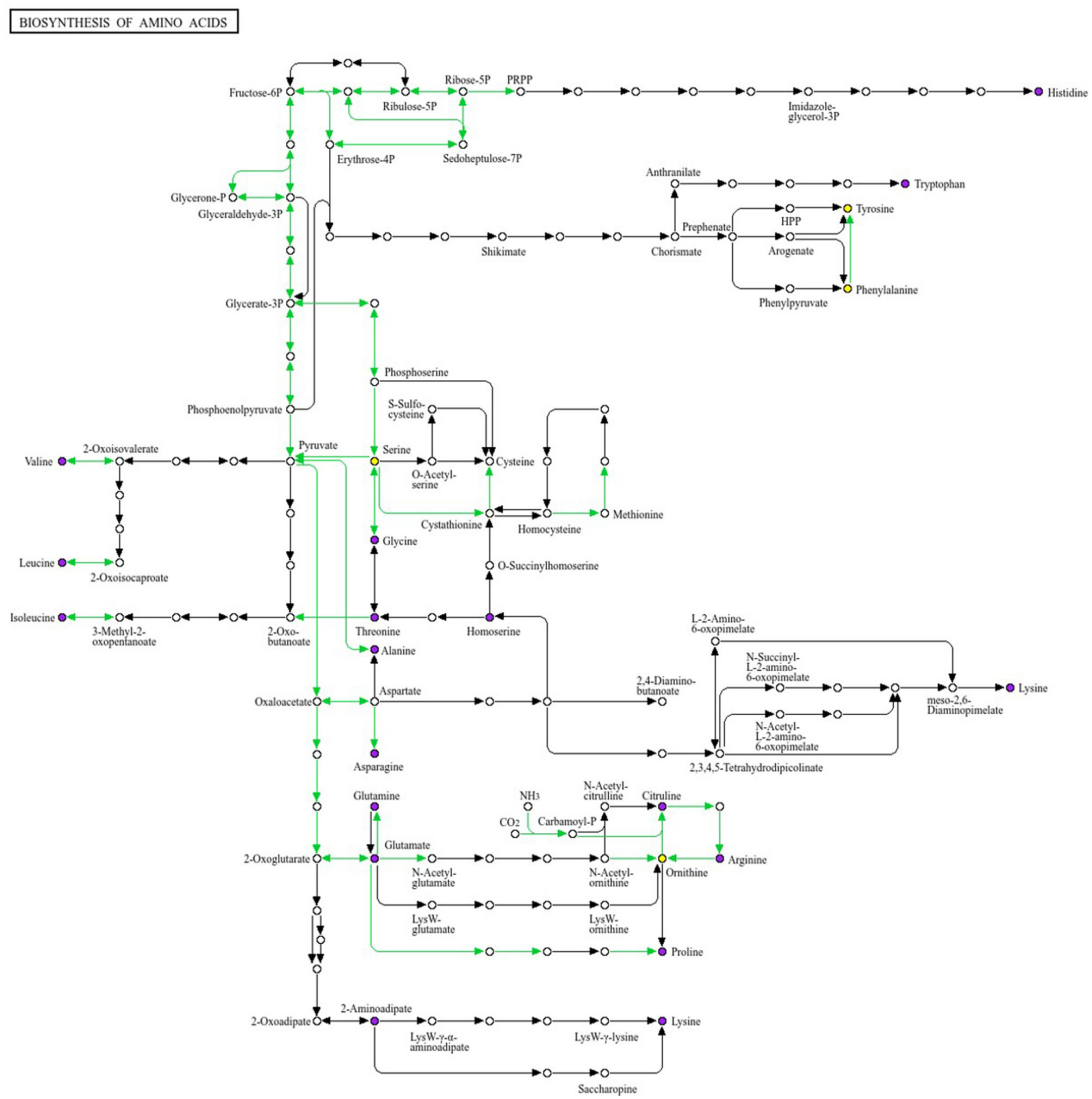
Mapping of the differential metabolites to the KEGG amino acid biosynthetic pathway (map01230). We selected the pathway with the largest number of enriched entries. This pathway diagram illustrates the modular architecture of the biosynthesis routes for 20 amino acids. Colored nodes denote metabolites identified as differentially abundant. In the between-group comparison, tryptophan,tyrosine, and glutamine show upward trends within the pathway, whereas valine, leucine and ornithine show downward trends. Differential abundance was defined using VIP > 1.0, |log_2_FC| ≥ 0.5, and FDR < 0.05 as criteria. KEGG enrichment analysis identified amino acid biosynthesis as the most enriched pathway.

Differential metabolites (VIP > 1.0, |log_2_FC| ≥ 0.5, FDR < 0.05) were subjected to KEGG enrichment analysis and then mapped to the amino acid biosynthesis pathway (KEGG map01230), which represented the most enriched pathway in this study (Figure 9). This pathway links glycolysis/TCA intermediates (3-phosphoglycerate, pyruvate, oxaloacetate, 2-oxoglutarate) to branches for aromatic amino acids (tryptophan, tyrosine, phenylalanine), branched-chain amino acids (BCAAs) (valine, leucine, isoleucine), and the glutamate/glutamine–ornithine/arginine–proline axis. In Group A (obesity), the abundance of tryptophan, tyrosine, isoleucine, and glutamine was elevated, whereas that of phenylalanine, valine, leucine, and ornithine was reduced within the pathway. This pattern was suggestive of an imbalanced carbon flow at the shikimate/chorismate node (reflected in Trp/Tyr upregulation with Phe downregulation), persistent suppression of the BCAA module (downregulation of Val/Leu), and a potential bottleneck at the urea cycle interface (downregulation of ornithine), with compensatory nitrogen buffering (glutamine upregulation). In Group B (obesity), the contents of tyrosine, phenylalanine, and glutamate were increased, whereas those of tryptophan, valine, and leucine were decreased, indicating that the Tyr/Phe arm was activated with concurrent Trp constraint and recurrent BCAA attenuation.

BCAA biosynthetic/turnover signals were consistently downregulated in both obesity groups (A and B), whereas aromatic branches diverged. Together, these pathway-level changes corroborated the targeted quantitation results and indicate that amino acid metabolism undergoes reprogramming in obesity, involving redistribution at the aromatic branch point and remodeling of the glutamate–ornithine axis. These findings provide mechanistic insights for guiding stratified nutritional or microbiota-directed interventions targeting BCAA and aromatic amino acid metabolism in obesity subtypes.

Metabolomics studies have shown that the serum levels of key amino acids and small-molecule metabolites, including carnosine, 3-methylhistidine, creatinine, ornithine, histidine, citrulline, cystine, glycine, and serine, are significantly altered in individuals with obesity (19). Elevated creatinine abundance may reflect obesity-related changes in muscle metabolism or a potential burden on renal function. The identification of creatinine, a product of muscle metabolism, as an obesity-specific marker suggests that kidney impairment may be an early indicator in the development of obesity, highlighting the importance of prompt and regular renal function monitoring in patients with this condition. The accumulation of adipose tissue in obesity may induce localized inflammatory responses, making carnosine a potential obesity-specific indicator. 3-Methylhistidine has notable blood lipid-lowering effects, thereby influencing lipid metabolism, and also participates in the regulation of thyroid function. Ornithine helps reduce cholesterol levels and serves as an antagonistic factor during obesity progression, alongside participating in primary bile acid metabolism and detoxification. Its elevated expression in obese individuals may be related to diet or its induction through fat accumulation. Histidine is an essential amino acid with a dual role in modulating inflammation and promoting vasodilation. It has strong regulatory effects on obesity, which provide valuable insights into the prevention of cardiovascular diseases and obesity-related complications. Citrulline regulates physiological functions and helps manage blood glucose and lipid levels. Cystine neutralizes toxins and promotes cellular oxidation, thereby influencing metabolic processes involved in obesity.

## 4 Discussion

The human gut harbors a vast microbial ecosystem, collectively known as the gut microbiota. Over 99% of the human gut microbiota consists of the phyla Firmicutes, Actinobacteria, Bacteroidetes, and Proteobacteria (20). In this study, we found that the abundance of Bacteroidetes was decreased, while that of Firmicutes was increased in patients with obesity. As noted by Patrice et al. (21), short-chain fatty acids(SCFAs) play a crucial role in gut microbiota metabolism. Gut dysbiosis leads to an increase in short-chain fatty acid production. Reduced Bacteroidetes abundance is associated with decreased production of acetate and propionate, which reduces the ability of the host to break down dietary fiber. Meanwhile, elevated Firmicutes abundance enhances butyrate production, which accelerates fat breakdown and energy absorption. Pinart et al. (22) showed that under similar conditions, germ-free mice that received gut microbiota from wild-type mice displayed a 60% increase in body fat. Furthermore, when gut microbiota from obese human adults was transplanted into germ-free mice, the mice also experienced an increase in body weight. DeGroot et al. (23) found that obese populations exhibit a higher abundance of bacteria that are efficient at energy capture, such as *Lactobacillus, Escherichia coli*, and *Bacteroides*. Furthermore, the abundance of *Bifidobacterium*, which can exert positive effects on gut function, was reported to be lower in obese than in non-obese individuals. Furthermore, the Firmicutes/Bacteroidetes ratio in obese populations is higher than that in normal controls, leading to metabolic disorders related to fat deposition in the host.

In this study, metabolomic analysis of patients with obesity showed that several differential metabolites were linked to aromatic amino acids and branched-chain amino acids (BCAAs), supporting an interaction between the gut microbiota and host amino-acid metabolism. The findings indicate that the microbiota exerts a substantial influence on circulating BCAA levels (valine, leucine, isoleucine); shifts in community structure—particularly the reduced abundance of *Bacteroides* observed in obesity—may underlie the elevated serum concentrations of aromatic amino acids and BCAAs. Moreover, glutamate, an amino acid that clearly distinguished obese patients from healthy controls, was positively correlated with the genus *Ruminococcus* and negatively correlated with glutamine. This further suggests that the abundance of specific taxa is associated with circulating amino-acid levels and may directly participate in amino-acid metabolism. Serum glutamate also showed a negative correlation with *Bacteroides*, implying that depletion of *Bacteroides* in obesity may contribute to increased glutamate levels. Taken together, these results indicate that amino-acid metabolism by specific gut microbes may modulate circulating amino acids linked to obesity and its metabolic complications.

Liu et al. (24) analyzed the correlation between gut microbiota composition and metabolite profiles in obese patients and found that tyrosine, phenylalanine, and glutamate abundance was closely related to changes in the microbiome in obesity. The authors further showed that changes in the gut microbiota, particularly a reduction in *Bacteroides* species in obese individuals, were associated with elevated concentrations of aromatic amino acids (tyrosine, phenylalanine, tryptophan) and BCAAs (leucine, isoleucine, valine) in the circulation. This shows that gut microbiota synergistically contribute to the development of obesity through their influence on host metabolic processes.

Combined analysis revealed that specific bacterial genera, such as *Bacteroides*, in the obese population were significantly correlated with amino acid metabolite contents, suggesting that the microbiota may directly or indirectly regulate host amino acid metabolic pathways (25). Compared with lean individuals, the gut microbiota in individuals with obesity showed significant enrichment of pathways related to amino acid metabolism, such as the biosynthesis of phenylalanine, tyrosine, and tryptophan, as well as glutamine and glutamate transport systems. Conversely, there was a reduction in the abundance of microbial genes associated with the degradation of valine, leucine, and isoleucine. This indicates that the gut microbiota in obese individuals has an elevated potential for synthesizing aromatic amino acids and BCAAs. These data suggest that the gut microbiota in the obese population not only enhances carbohydrate utilization but also potentially promotes the synthesis of pro-inflammatory factors and aromatic and BCAAs. This microbiota-metabolite network provides a theoretical basis for the development of metabolism-targeted therapeutic strategies, such as probiotics and dietary interventions, aimed at microbiota modulation.

Our data further showed that Enterobacteriales were markedly enriched in obese individuals, likely due to chronic inflammation, whereas the abundance of Clostridiales, considered a beneficial bacterial group, was reduced in obesity. 3-Methylhistidine, a specific biomarker of muscle protein breakdown, is also involved in inflammation and thyroid function regulation. Given the rising recognition of depression as a major driver of obesity, 3-methylhistidine may additionally serve as an emotion-related indicator. The increase in its abundance in obesity could promote a compensatory decrease in that of Firmicutes and Clostridiales. Carnosine exhibits antioxidant, anti-glycation, and anti-inflammatory properties. Chronic low-grade inflammation is a central feature of obesity, especially at the intestinal level. The elevated levels of carnosine observed in obesity likely reflect such inflammatory responses. Furthermore, the abundance of Selenomonadales was significantly increased in obese individuals, potentially contributing to altered gut permeability (“leaky gut”) through their metabolic products, further promoting low-grade chronic inflammation. Moreover, histidine concentrations were found to be significantly decreased in individuals with obesity compared to those in normal-weight controls. Reduced histidine levels have been associated with enhanced inflammation and disrupted nitrogen metabolism. Bifidobacteriales may influence histidine levels, thereby affecting gut barrier function and perpetuating chronic inflammation, ultimately contributing to the development of obesity (26, 27).

### 4.1 Clinical implications and potential directions for intervention

Our results suggested that the gut microbiota and metabolomic profiles may serve as early diagnostic markers and therapeutic targets for obesity-related diseases (27). Body weight can be regulated by modulating gut microbiota composition and serumamino acid levels, potentially preventing the development of metabolic diseases (28). Similarly, dietary adjustments that modulate microbiota composition may also lead to weight loss. Future research could explore interventions for obesity through precision nutrition, fecal microbiota transplantation (FMT), or synthetic microbiota approaches to modulate the microbiome.

## 5 Limitations and future prospects

This study has several limitations. First, the relatively modest sample size (*n* = 60) may limit the generalizability of our findings. Second, although strict inclusion criteria were applied—such as the exclusion of recent antibiotic or probiotic users—we did not systematically record participants' dietary intake, physical activity levels, or other detailed lifestyle habits. Moreover, potential confounding factors such as sex, diet, and medication use, which are known to influence gut microbiota and metabolic profiles, were not fully controlled or examined through subgroup analyses. These uncontrolled variables may have contributed to inter-individual variability and influenced the observed microbial and metabolomic patterns. Additionally, the use of 16S rRNA sequencing limits taxonomic resolution at the species level and lacks the capacity for robust functional prediction. Future research should employ metagenomic sequencing and multi-omics approaches, alongside larger, well-stratified cohorts and intervention trials to validate our findings, control for lifestyle-related confounders, and explore underlying causal relationships.

## 6 Conclusions

From phylum to species, obese individuals exhibited marked dysbiosis, characterized by enrichment of Proteobacteria and related clades (e.g., Gammaproteobacteria, Enterobacteriales, and Enterobacteriaceae) and shifts in the composition of some putatively beneficial taxonomic groups, such as *Escherichia–Shigella* lineages and several species. These taxonomic groups may serve as biomarkers of obesity and potential therapeutic targets. Untargeted metabolomics analysis indicated that differential metabolites were concentrated in amino acids, fatty acids, and carboxylic acids. Targeted amino acid profiling further confirmed that the contents of carnosine, creatinine, ornithine, citrulline, glycine, cystine, and serine underwent significant alterations in obesity, implicating amino acid metabolic imbalance in this condition. Integration of microbiome and metabolome data indicated that obesity-related disturbances primarily involve dysregulated lipid metabolism and heightened inflammatory responses, with shifts in amino acid metabolism potentially contributing to obesity *via* the modulation of inflammation and energy metabolism.

## Data Availability

The original contributions presented in the study are publicly available. This data can be found here: https://www.ncbi.nlm.nih.gov/bioproject/PRJNA1336992.
