# Supplementary material for: Integrated gut microbiota and metabolomic profiling reveals key associations between amino acid levels and gut microbial composition in patients with obesity
Source: Front Nutr. 2025 Oct 24;12:1648469. doi: 10.3389/fnut.2025.1648469 (PMC12591972; doi:10.3389/fnut.2025.1648469)
Supplement: Supplementary file 1 [file Table_1.DOCX]

Supplementary Material

# Supplementary Data

## Table 1. Relative abundance of bacterial classes in obese patients and normal group

| Class | hz（obese patients） | zc（normal group） |
| --- | --- | --- |
| Clostridia | 43.637 | 53.251 |
| Gammaproteobacteria | 20.473 | 8.115 |
| Bacteroidia | 10.758 | 14.468 |
| Actinobacteria | 7.322 | 10.264 |
| Negativicutes | 5.128 | 3.472 |
| Bacilli | 3.473 | 4.315 |
| Erysipelotrichia | 4.051 | 1.681 |
| Verrucomicrobiae | 2.9 | 1.822 |
| Coriobacteriia | 1.196 | 1.154 |
| Deltaproteobacteria | 0.168 | 0.287 |
| Other | 0.177 | 0.334 |
| Unclassified | 0.719 | 0.838 |

## Table 2. Relative abundance of bacterial families in obese patients and normal group

| Family | hz（obese patients） | zc（normal group） |
| --- | --- | --- |
| Lachnospiraceae | 24.404 | 23.988 |
| Ruminococcaceae | 17.942 | 26.525 |
| Enterobacteriaceae | 19.612 | 7.424 |
| Bifidobacteriaceae | 7.209 | 10.119 |
| Bacteroidaceae | 6.571 | 7.935 |
| Veillonellaceae | 3.391 | 2.588 |
| Erysipelotrichaceae | 4.051 | 1.681 |
| Akkermansiaceae | 2.897 | 1.818 |
| Prevotellaceae | 1.361 | 2.745 |
| Streptococcaceae | 1.545 | 1.527 |
| Other | 9.848 | 11.973 |
| Unclassified | 1.17 | 1.677 |

## Table 3. Relative abundance of bacterial phylum in obese patients and normal group

| Phylum | hz（obese patients） | zc（normal group） |
| --- | --- | --- |
| Firmicutes | 56.485 | 62.927 |
| Proteobacteria | 20.694 | 8.463 |
| Bacteroidetes | 10.758 | 14.469 |
| Actinobacteria | 8.522 | 11.425 |
| Verrucomicrobia | 2.9 | 1.822 |
| Fusobacteria | 0.061 | 0.085 |
| Euryarchaeota | 0.007 | 0.071 |
| Tenericutes | 0.005 | 0.047 |
| Patescibacteria | 0.028 | 0.017 |
| Acidobacteria | 0.008 | 0.02 |
| Other | 0.03 | 0.046 |
| Unclassified | 0.503 | 0.607 |

## Table 4. Relative abundance of bacterial orders in obese patients and normal group

| Order | hz（obese patients） | zc（normal group） |
| --- | --- | --- |
| Clostridiales | 43.637 | 53.25 |
| Enterobacteriales | 19.612 | 7.424 |
| Bacteroidales | 10.725 | 14.426 |
| Bifidobacteriales | 7.209 | 10.119 |
| Selenomonadales | 5.128 | 3.472 |
| Lactobacillales | 3.432 | 4.286 |
| Erysipelotrichales | 4.051 | 1.681 |
| Verrucomicrobiales | 2.898 | 1.818 |
| Coriobacteriales | 1.196 | 1.154 |
| Betaproteobacteriales | 0.652 | 0.467 |
| Other | 0.711 | 1.034 |
| Unclassified | 0.751 | 0.869 |

## Table 5. Relative abundance of bacterial genera in obese patients and normal group

| Genus | hz（obese patients） | zc（normal group） |
| --- | --- | --- |
| Faecalibacterium | 9.147 | 13.858 |
| Escherichia-Shigella | 14.939 | 6.223 |
| Bifidobacterium | 7.195 | 10.102 |
| Bacteroides | 6.571 | 7.935 |
| Blautia | 7.829 | 5.363 |
| Subdoligranulum | 2.821 | 3.29 |
| Akkermansia | 2.897 | 1.818 |
| Klebsiella | 3.016 | 0.783 |
| Ruminococcus_torques_group | 2.066 | 1.592 |
| Erysipelotrichaceae_UCG-003 | 2.145 | 0.841 |
| Other | 33.886 | 36.456 |
| Unclassified | 7.488 | 11.741 |

## Table 6. Relative abundance of bacterial species in obese patients and normal group

| Species | hz（obese patients） | zc（normal group） |
| --- | --- | --- |
| Ruminococcus_sp_Marseille-P328 | 0.966 | 1.415 |
| Phascolarctobacterium_faecium | 1.458 | 0.728 |
| Bifidobacterium_longum_subsp_longum | 1.03 | 0.974 |
| Bacteroides_uniformis | 0.656 | 1.131 |
| Citrobacter_freundii | 1.355 | 0.265 |
| Bacteroides_fragilis | 1.258 | 0.109 |
| Parabacteroides_distasonis | 1.028 | 0.305 |
| Bacteroides_caccae | 0.835 | 0.412 |
| Bacteroides_coprocola_DSM_17136 | 0.234 | 0.574 |
| Bacteroides_stercoris_ATCC_43183 | 0.434 | 0.338 |
| Other | 3.767 | 5.975 |
| Unclassified | 86.978 | 87.775 |

## Table 7: AUC of gut microbiota at the phylum level

| Factor | AUC | 95% CI (AUC) | best_thresholds (specificities,sensitivities) |
| --- | --- | --- | --- |
| Proteobacteria | 0.709 | 0.569~0.848 | 5.7213 (0.775,0.7) |
| Actinobacteria | 0.659 | 0.518~0.799 | 1.62495 (0.375,0.95),(0.425,0.9) |
| Fusobacteria | 0.704 | 0.571~0.837 | 0.0122 (0.65,0.85) |
| Tenericutes | 0.735 | 0.603~0.867 | 0.00165 (0.65,0.85) |

## Table 8 :AUC of gut microbiota at the class level

| Factor | AUC | 95% CI (AUC) | best_thresholds (specificities,sensitivities) |
| --- | --- | --- | --- |
| Clostridia | 0.68 | 0.533~0.827 | 58.0762 (0.825,0.55) |
| Gammaproteobacteria | 0.712 | 0.573~0.852 | 5.29325 (0.775,0.7) |
| Erysipelotrichia | 0.614 | 0.471~0.757 | 4.4134 (0.3,1) |
| Fusobacteriia | 0.704 | 0.571~0.837 | 0.0122 (0.65,0.85) |
| Alphaproteobacteria | 0.668 | 0.533~0.803 | 0.0244 (0.475,1) |
| Mollicutes | 0.735 | 0.603~0.867 | 0.00165 (0.65,0.85) |

## Table 9 : AUC of gut microbiota at the class level

| Factor | AUC | 95% CI (AUC) | best_thresholds (specificities,sensitivities) |
| --- | --- | --- | --- |
| Clostridiales | 0.68 | 0.533~0.827 | 58.0762 (0.825,0.55) |
| Enterobacteriales | 0.734 | 0.597~0.871 | 4.454 (0.75,0.7) |
| Erysipelotrichales | 0.614 | 0.471~0.757 | 4.4134 (0.3,1) |
| Betaproteobacteriales | 0.67 | 0.528~0.812 | 0.4011 (0.775,0.65) |
| Fusobacteriales | 0.704 | 0.571~0.837 | 0.0122 (0.65,0.85) |
| Bacillales | 0.658 | 0.506~0.81 | 0.0317 (0.425,0.9) |
| Mollicutes_RF39 | 0.737 | 0.608~0.867 | 0.00155 (0.8,0.7) |

## Table 10 : AUC of gut microbiota at the order level

| Factor | AUC | 95% CI (AUC) | best_thresholds (specificities,sensitivities) |
| --- | --- | --- | --- |
| Clostridiales | 0.68 | 0.533~0.827 | 58.0762 (0.825,0.55) |
| Enterobacteriales | 0.734 | 0.597~0.871 | 4.454 (0.75,0.7) |
| Erysipelotrichales | 0.614 | 0.471~0.757 | 4.4134 (0.3,1) |
| Betaproteobacteriales | 0.67 | 0.528~0.812 | 0.4011 (0.775,0.65) |
| Fusobacteriales | 0.704 | 0.571~0.837 | 0.0122 (0.65,0.85) |
| Bacillales | 0.658 | 0.506~0.81 | 0.0317 (0.425,0.9) |
| Mollicutes_RF39 | 0.737 | 0.608~0.867 | 0.00155 (0.8,0.7) |

## Table 11 : AUC of gut microbiota at the genus level

| Factor | AUC | 95% CI (AUC) | best_thresholds (specificities,sensitivities) |
| --- | --- | --- | --- |
| Escherichia-Shigella | 0.71 | 0.565~0.855 | 1.47345 (0.75,0.7) |
| Bifidobacterium | 0.658 | 0.513~0.802 | 1.7726 (0.625,0.7) |
| Blautia | 0.581 | 0.435~0.728 | 11.2566 (0.3,1) |
| Klebsiella | 0.688 | 0.537~0.838 | 0.2538 (0.725,0.7) |
| Megamonas | 0.706 | 0.537~0.875 | 0.04015 (0.9,0.6) |
| Alistipes | 0.759 | 0.631~0.886 | 0.9446 (0.8,0.7) |
| Dialister | 0.732 | 0.607~0.858 | 0.0527 (0.525,1) |
| Citrobacter | 0.669 | 0.528~0.809 | 0.17545 (0.5,0.8),(0.55,0.75) |
| Ruminococcaceae_UCG-014 | 0.841 | 0.744~0.938 | 0.0482 (0.675,1) |
| Roseburia | 0.774 | 0.658~0.89 | 0.3293 (0.625,0.9) |
| Lachnoclostridium | 0.711 | 0.563~0.859 | 0.2083 (0.7,0.7) |
| Ruminococcaceae_UCG-002 | 0.682 | 0.544~0.821 | 0.4234 (0.675,0.7) |
| Christensenellaceae_R-7_group | 0.761 | 0.642~0.88 | 0.02365 (0.475,1) |
| Romboutsia | 0.77 | 0.652~0.888 | 0.0257 (0.6,0.95) |
| Lactococcus | 0.832 | 0.732~0.933 | 0.00995 (0.6,1),(0.65,0.95) |
| Coprococcus_3 | 0.664 | 0.521~0.806 | 0.2201 (0.75,0.55) |
| Barnesiella | 0.812 | 0.697~0.927 | 0.0109 (0.675,0.95) |
| Ruminococcaceae_UCG-005 | 0.668 | 0.531~0.804 | 0.00795 (0.325,1),(0.375,0.95) |
| Prevotella_2 | 0.832 | 0.732~0.932 | 0.00745 (0.675,0.9) |
| Ruminococcus_1 | 0.832 | 0.73~0.934 | 0.0293 (0.775,0.85) |
| Clostridium_sensu_stricto_1 | 0.742 | 0.618~0.866 | 0.0211 (0.675,0.85) |
| Raoultella | 0.703 | 0.557~0.849 | 0.0104 (0.8,0.6) |
| Ruminococcaceae_UCG-004 | 0.711 | 0.572~0.851 | 0.07925 (0.775,0.6) |
| Erysipelatoclostridium | 0.769 | 0.636~0.901 | 0.00965 (0.775,0.7) |
| Desulfovibrio | 0.744 | 0.62~0.869 | 0.00985 (0.625,0.85) |
| Paraprevotella | 0.732 | 0.6~0.865 | 0.01615 (0.775,0.7) |
| Ruminiclostridium_5 | 0.705 | 0.573~0.837 | 0.03575 (0.475,0.95) |
| Veillonella | 0.789 | 0.673~0.904 | 0.0114 (0.525,1) |
| Lachnospiraceae_NK4A136_group | 0.819 | 0.711~0.926 | 0.02335 (0.575,0.9),(0.625,0.85),(0.825,0.65) |
| Eubacterium_ruminantium_group | 0.851 | 0.756~0.945 | 0.01175 (0.65,1) |
| Lachnospira | 0.728 | 0.593~0.863 | 0.012 (0.55,0.8),(0.65,0.7),(0.7,0.65),(0.85,0.5),(0.9,0.45) |
| Eubacterium_eligens_group | 0.69 | 0.554~0.826 | 0.00525 (0.45,0.95) |
| Negativibacillus | 0.69 | 0.556~0.824 | 0.0044 (0.575,0.8) |
| Burkholderia-Caballeronia-Paraburkholderia | 0.664 | 0.515~0.812 | 0.01465 (0.625,0.65) |
| Rikenellaceae_RC9_gut_group | 0.864 | 0.773~0.956 | 0.00135 (0.7,1) |
| Catenibacterium | 0.909 | 0.825~0.993 | 0.0022 (0.825,0.95),(0.925,0.85) |
| Eggerthella | 0.7 | 0.55~0.85 | 0.00835 (0.725,0.7) |
| Eisenbergiella | 0.78 | 0.665~0.895 | 0.0045 (0.6,0.95),(0.7,0.85) |
| Peptoclostridium | 0.872 | 0.778~0.967 | 9e-04 (0.85,0.9) |
| Rothia | 0.657 | 0.514~0.8 | 0.00625 (0.425,0.9) |
| Ruminococcaceae_UCG-010 | 0.806 | 0.695~0.918 | 0.0013 (0.625,0.95),(0.725,0.85) |
| Pseudomonas | 0.699 | 0.563~0.834 | 0.00685 (0.475,0.95) |
| Coprobacillus | 0.731 | 0.601~0.861 | 0.00165 (0.675,0.9) |
| Lachnospiraceae_UCG-010 | 0.757 | 0.634~0.88 | 0.01445 (0.675,0.85),(0.725,0.8) |
| Serratia | 0.622 | 0.476~0.769 | 0.0212 (0.425,0.9) |
| Odoribacter | 0.726 | 0.593~0.858 | 0.023 (0.775,0.7) |
| Clostridium_innocuum_group | 0.632 | 0.489~0.776 | 0.0057 (0.55,0.75) |
| Peptoniphilus | 0.688 | 0.544~0.832 | 0.0022 (0.65,0.75) |
| Ruminiclostridium_6 | 0.764 | 0.634~0.895 | 0.0038 (0.8,0.7),(0.85,0.65) |
| Neisseria | 0.799 | 0.678~0.92 | 0.00375 (0.55,0.95),(0.65,0.85) |
| Flavonifractor | 0.679 | 0.543~0.816 | 0.00295 (0.475,0.95) |
| Lautropia | 0.698 | 0.56~0.835 | 0.00185 (0.625,0.8) |
| Providencia | 0.762 | 0.684~0.841 | 5e-04 (0.525,1) |
| Olsenella | 0.672 | 0.534~0.811 | 0.0016 (0.625,0.8) |
| Sellimonas | 0.784 | 0.669~0.898 | 0.0013 (0.525,1) |
| Holdemania | 0.673 | 0.534~0.813 | 0.00435 (0.575,0.8) |
| Lysinibacillus | 0.73 | 0.614~0.846 | 0.0065 (0.4,1),(0.45,0.95) |
| Leptotrichia | 0.731 | 0.594~0.869 | 0.0026 (0.675,0.85),(0.725,0.8),(0.775,0.75) |
| Prevotella | 0.806 | 0.695~0.918 | 0.00155 (0.625,0.95) |
| Abiotrophia | 0.698 | 0.559~0.836 | 0.00135 (0.5,0.85),(0.55,0.8),(0.6,0.75) |
| Sutterella | 0.654 | 0.504~0.803 | 0.0088 (0.9,0.4) |
| Anaerococcus | 0.674 | 0.558~0.79 | 0.00055 (0.5,0.85) |
| Pediococcus | 0.716 | 0.588~0.844 | 0.00155 (0.875,0.55) |
| Marvinbryantia | 0.722 | 0.582~0.863 | 0.003 (0.775,0.65) |
| Parvimonas | 0.734 | 0.605~0.862 | 0.0025 (0.55,0.9) |
| CAG-352 | 0.625 | 0.516~0.734 | 7e-04 (0.95,0.3) |

## Table 12 : AUC of gut microbiota at the species level

| Factor | AUC | 95% CI (AUC) | best_thresholds (specificities,sensitivities) |
| --- | --- | --- | --- |
| Citrobacter_freundii | 0.669 | 0.528~0.809 | 0.17545 (0.5,0.8),(0.55,0.75) |
| Bacteroides_fragilis | 0.733 | 0.593~0.873 | 0.0192 (0.85,0.6) |
| Bacteroides_uniformis | 0.74 | 0.614~0.866 | 0.0721 (0.5,1) |
| Parabacteroides_distasonis | 0.61 | 0.466~0.754 | 0.72375 (0.325,0.95),(0.525,0.75) |
| Bacteroides_stercoris_ATCC_43183 | 0.721 | 0.592~0.851 | 0.0334 (0.425,1) |
| Bacteroides_coprocola_DSM_17136 | 0.734 | 0.608~0.859 | 0.0108 (0.55,1) |
| Lactobacillus_gasseri | 0.844 | 0.752~0.935 | 0.01155 (0.7,0.95) |
| Bacteroides_eggerthii | 0.796 | 0.686~0.907 | 0.0083 (0.625,1) |
| Lactobacillus_salivarius | 0.826 | 0.721~0.932 | 0.01265 (0.75,0.95) |
| Parabacteroides_merdae | 0.666 | 0.531~0.802 | 0.07625 (0.55,0.85) |
| Lactococcus_garvieae_subsp_garvieae | 0.822 | 0.718~0.926 | 0.0079 (0.7,0.95) |
| Streptococcus_pneumoniae | 0.672 | 0.528~0.817 | 0.07025 (0.575,0.85) |
| Bacteroides_sp | 0.769 | 0.652~0.886 | 0.0038 (0.525,1) |
| Lactobacillus_ruminis | 0.838 | 0.736~0.939 | 0.0037 (0.725,1) |
| Alistipes_shahii_WAL_8301 | 0.731 | 0.606~0.857 | 0.01505 (0.55,0.9) |
| Clostridium_perfringens_CPE_str_F4969 | 0.804 | 0.694~0.914 | 0.0048 (0.65,1) |
| Faecalibacterium_prausnitzii | 0.757 | 0.635~0.879 | 0.0028 (0.45,1) |
| Ruminococcus_bicirculans | 0.879 | 0.792~0.965 | 0.009 (0.775,0.95),(0.825,0.9) |
| Desulfovibrio_piger | 0.711 | 0.579~0.843 | 0.00155 (0.45,0.95),(0.6,0.8) |
| Alistipes_indistinctus_YIT_12060 | 0.696 | 0.565~0.828 | 0.00165 (0.425,0.95),(0.525,0.85) |
| Ruminococcus_callidus_ATCC_27760 | 0.742 | 0.606~0.878 | 0.00705 (0.75,0.75) |
| Lactococcus_lactis | 0.754 | 0.616~0.891 | 0.00615 (0.825,0.65),(0.875,0.6) |
| Porphyromonas_sp_HMSC065F10 | 0.812 | 0.737~0.888 | 5e-04 (0.625,1) |
| Alistipes_obesi | 0.709 | 0.573~0.845 | 0.02325 (0.775,0.65) |
| Fusobacterium_mortiferum_ATCC_9817 | 0.667 | 0.529~0.805 | 0.00135 (0.625,0.8) |
| Serratia_marcescens | 0.622 | 0.476~0.769 | 0.0212 (0.425,0.9) |
| Odoribacter_splanchnicus_DSM_20712 | 0.732 | 0.601~0.863 | 0.023 (0.775,0.7) |
| Prevotellamassilia_timonensis | 0.764 | 0.661~0.866 | 0.00055 (0.6,0.95) |
| Emergencia_timonensis | 0.347 | 0.259~0.434 | -Inf (0,1),(1,0) |
| Eubacterium_sp_1-5 | 0.709 | 0.574~0.844 | 0.00155 (0.75,0.75) |
| Olsenella_sp_kh2p3 | 0.73 | 0.595~0.865 | 0.00135 (0.675,0.8),(0.725,0.75) |
| Eubacterium_siraeum_DSM_15702 | 0.81 | 0.693~0.927 | 0.00135 (0.8,0.8) |
| Bacteroides_nordii | 0.645 | 0.503~0.787 | 0.00155 (0.675,0.65) |
| Pediococcus_pentosaceus | 0.716 | 0.588~0.844 | 0.00155 (0.875,0.55) |
| bacterium_NLAE-zl-G241 | 0.678 | 0.536~0.819 | 0.00215 (0.5,0.85),(0.7,0.65) |
| Alistipes_inops | 0.651 | 0.538~0.764 | 7e-04 (0.95,0.35) |
| Anaerococcus_sp_HMSC068A02 | 0.332 | 0.245~0.419 | -Inf (0,1),(1,0) |
| Coprobacillus_cateniformis_JCM_10604 | 0.658 | 0.522~0.794 | 7e-04 (0.75,0.55) |
| Bacillus_nealsonii | 0.358 | 0.25~0.465 | -Inf (0,1),(1,0) |
| iron-reducing_bacterium_enrichment_culture_clone_HN70 | 0.55 | 0.483~0.617 | 9e-04 (1,0.1) |
